# Specialized Metabolites from the Allelopathic Plant *Retama raetam* as Potential Biopesticides

**DOI:** 10.3390/toxins14050311

**Published:** 2022-04-28

**Authors:** Gabriele Soriano, Claudia Petrillo, Marco Masi, Mabrouka Bouafiane, Aminata Khelil, Angela Tuzi, Rachele Isticato, Mónica Fernández-Aparicio, Alessio Cimmino

**Affiliations:** 1 Department of Chemical Sciences, University of Naples Federico II, 80126 Naples, Italy; gabriele.soriano@unina.it (G.S.); angela.tuzi@unina.it (A.T.); alessio.cimmino@unina.it (A.C.); 2 Department of Biology, University of Naples Federico II, 80126 Naples, Italy; isticato@unina.it; 3 Laboratoire de Protection des Ecosystèmes en Zones Arides et Semi-Arides, Universit’e Kasdi Merbah-Ouargla, Ouargla 30000, Algeria; bouafiane-mabrouka@univ-eloued.dz (M.B.); aminatakhelil@yahoo.fr (A.K.); 4 Department of Agronomy, Faculty of Life and Natural Sciences, University of El Oued, El Oued 39000, Algeria; 5 Department of Plant Breeding, Institute for Sustainable Agriculture (IAS), CSIC, Avenida Menéndez Pidal s/n, 14004 Cόrdoba, Spain; monica.fernandez@ias.csic.es

**Keywords:** biocontrol, *Retama raetam*, *Stemphylium vesicarium*, *Orobanche cumana*, laburnetin, ephedroidin

## Abstract

To cope with the rising food demand, modern agriculture practices are based on the indiscriminate use of agrochemicals. Although this strategy leads to a temporary solution, it also severely damages the environment, representing a risk to human health. A sustainable alternative to agrochemicals is the use of plant metabolites and plant-based pesticides, known to have minimal environmental impact compared to synthetic pesticides. *Retama raetam* is a shrub growing in Algeria’s desert areas, where it is commonly used in traditional medicine because of its antiseptic and antipyretic properties. Furthermore, its allelopathic features can be exploited to effectively control phytopathogens in the agricultural field. In this study, six compounds belonging to isoflavones and flavones subgroups have been isolated from the *R. raetam* dichloromethane extract and identified using spectroscopic and optical methods as alpinumisoflavone, hydroxyalpinumisoflavone, laburnetin, licoflavone C, retamasin B, and ephedroidin. Their antifungal activity was evaluated against the fungal phytopathogen *Stemphylium vesicarium* using a growth inhibition bioassay on PDA plates. Interestingly, the flavonoid laburnetin, the most active metabolite, displayed an inhibitory activity comparable to that exerted by the synthetic fungicide pentachloronitrobenzene, in a ten-fold lower concentration. The allelopathic activity of *R. raetam* metabolites against parasitic weeds was also investigated using two independent parasitic weed bioassays to discover potential activities on either suicidal stimulation or radicle growth inhibition of broomrapes. In this latter bioassay, ephedroidin strongly inhibited the growth of *Orobanche cumana* radicles and, therefore, can be proposed as a natural herbicide.

## 1. Introduction

Since the second half of the 20th century, the agricultural practices have been based on the uncontrolled use of chemical pesticides, and herbicides, to cope with the rising crop demands of an ever-growing human population [1,2]. As a consequence of the massive use of agrochemicals, harmful effects such as environmental pollution, human health threats, insect resistance to pesticides, parasitoids, and pollinators loss occurred [3,4,5]. Therefore, researchers are seeking alternative and eco-friendly solutions. Within this framework, plants are receiving increasing attention as remarkable sources of bioactive substances, due to their ability to synthesize an impressive variety of low molecular weight metabolites, often exploited as biocontrol agents in the agricultural field [4,6,7,8]. Interestingly, the release of these compounds can either be constitutive, or induced in response to a pathogenic attack [9], and their biosynthesis is closely related to the growth stage of the plant [10].

Plant diseases represent a considerable threat to agricultural production due to phytopathogenic fungi [11]. One of the most relevant fungal diseases is the brown spot of pear caused by *Stemphylium vesicarium* (Wallr.) E.G. Simmons, which every year leads to important economic losses in the European pear production areas. *S. vesicarium* has pathogenic effects on many other hosts, such as garlic, onion, and asparagus [12]. So far, the most effective method to control *S. vesicarium* spread is the preventive application of chemical fungicides such as dithiocarbamates or strobilurins [13].

Moreover, a large number of parasitic plants, such as broomrape (*Orobanche* and *Phelipanche* spp.), are adapted to infect crops in agriculture environments, depicting a serious threat to crop productivity by inducing severe yield losses [14]. For many crops, single methods of parasitic weed control are limited or non-existing, and thus integrated pest management systems appear the best solution to find effective, long-lasting, widely applicable, and environmentally benign methods for parasitic weed control. The identification of plant species as sources of allelopathic molecules against the parasitic weed lifecycle acting as either inducers of suicidal germination or inhibitors of radicle growth provide alternative methods for their use in integrated methods of parasitic weed control [15,16].

*Retama* spp. are perennial and unarmed shrubs from the Fabaceae family. They have been used traditionally for the treatment of different diseases in many parts of the Mediterranean Basin, especially in North Africa and the Middle East. In fact, they showed several biological activities, including antibacterial [17], anti-inflammatory [18], antioxidant [19], anti-proliferative [20], anti-ulcer [19], anti-viral [21], and hepatoprotective activities [22,23].

Among them, *Retama raetam* is mainly distributed in North Africa, including Morocco, Algeria, Tunisia, Libya, Egypt, Asia, and certain Middle Eastern countries, and widely used in the folk medicine, as a powder, an infusion, or decoction, to treat different diseases [10]. Within the potential applications, *R. raetam* has been employed to treat several disorders, including sore throat, skin diseases, fever, and inflammation [24], and it also displayed strong antioxidant, antimicrobial, hepatoprotective, and hypoglycemic activity [10]. According to most authors, *R. raetam* properties may be associated with its abundance in flavonoids, isoflavonoids, and alkaloids [24], which have also been related to the inhibition of a range of root pathogens and pests, ranging from bacteria to fungi and insects [25]. Furthermore, as reported by Chouikh et al. [26], this plant is an extraordinary source of various phenolic compounds, which are known to possess a strong antioxidant effect on free radicals and antibacterial effects. A recent study has shown that natural flavonoids from *R. raetam* exhibit important interaction with the active site of α-glucosidase, inspiring the development of a new drug with anti-diabetic activity [27].

Despite the numerous studies cited above, the antifungal activity against the fungal pathogen *S. vesicarium* has not been investigated. Moreover, the plant studied was collected in the Souf region located in the north-east of the Algerian Saharan desert, an extremely arid zone of great importance among medicinal plants [28,29,30,31]. Recently, researchers have highlighted the effectiveness of aqueous extracts of some Saharan plant species, including *R. raetam,* on seed germination or seedling growth of target species [32]. This result suggests that the plant can be a source of allelochemicals able to inhibit the radicle growth of parasitic weeds [15].

Thus, the main purpose of the present study was to further investigate the potential of the crude organic extract obtained from the *R. raetam* aerial part as a source of biopoesticides. Bioactivity-guided purification was performed using anti-fungal bioassays against the phytopathogenic fungus *S. vesicarium.* This is a traditional method used in natural product discovery that allows researchers to isolate the pure bioactive compounds from a complex mixture, such as plants’ organic extracts [33,34,35,36]. In particular, following the identification of a crude extract with promising biological activity, the next step is its (often multiple) consecutive bioactivity-guided fractionation until the pure bioactive compounds are isolated [37].

This manuscript reports the isolation of six metabolites identified by spectroscopic and chemical methods. Their potential antifungal activity against the phytopathogen *S. vesicarium* and the herbicidal activity against broomrapes (as inductors of suicidal germination and inhibitors of radicle growth), are also discussed.

## 2. Results and Discussion

**Figure 1:**
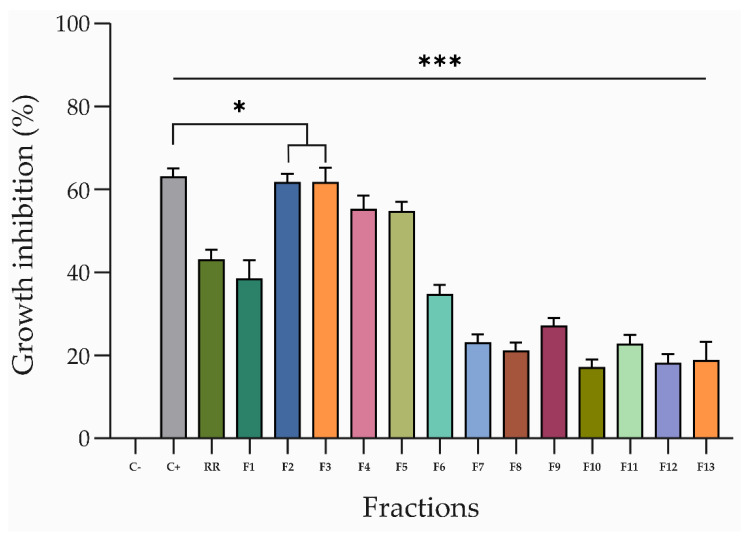
Inhibitory effect of the CH_2_Cl_2_ crude extract (RR) and the thirteen fractions obtained from the preliminary purification of RR, on the mycelium of *S. vesicarium*, at a concentration of 2 mg/mL (crude extract) and 250 μg/mL (fractions), respectively. The negative control was absolute methanol (C−), and the positive control was 200 μg/mL of PCNB. The fungal growth inhibition is represented as the percentage reduction of the fungal mycelia diameter in the treated plate compared to that in the control plate. All experiments were performed in triplicate with three independent trials. Data are presented as means ± standard deviation (*n* = 3) compared to the control. *** *p* < 0.0001 and * *p* < 0.05.

The dried aerial parts of *R. raetam* were extracted as detailed in the Materials and Methods Section. Bioactivity-guided purification was performed using anti-fungal bioassays against the phytopathogenic fungus *S. vesicarium*. The active CH_2_Cl_2_ extract was fractionated by CC, yielding 13 fractions (F1–13) with variable chemical profiles, which were evaluated for their antifungal activity against *S. vesicarium* (Figure 1).

**Figure 2:**
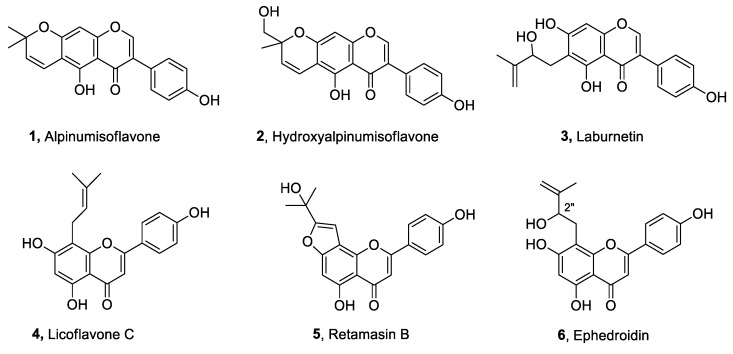
Chemical structures of specialized metabolites (**1**–**6**) isolated from *Retama raetam*.

Interestingly, the activity exhibited by the fractions F2–F5 (~60%) was stronger than that displayed by the total crude extract (~43%) and comparable to the commercial fungicide pentachloronitrobenzene (PCNB), used as a positive control (Figure 1). These latter fractions were further purified by combined column and TLCs (Scheme S1) to afford six pure metabolites (**1**–**6**, Figure 2), as described in the Materials and Methods Section. The first investigation of their ^1^H NMR and ESI MS spectra (Figures S1–S14) showed that they are isoflavones and flavones with different substitutions. They were identified by comparing their ^1^H NMR and MS data with those reported in the literature as alpinumisoflavone (**1**) [38,39,40], hydroxyalpinumisoflavone (**2**) [41], laburnetin (**3**) [42], licoflavone C (**4**) [43], retamasin B (**5**) [44], and ephedroidin (**6**) [41].

**Figure 3:**
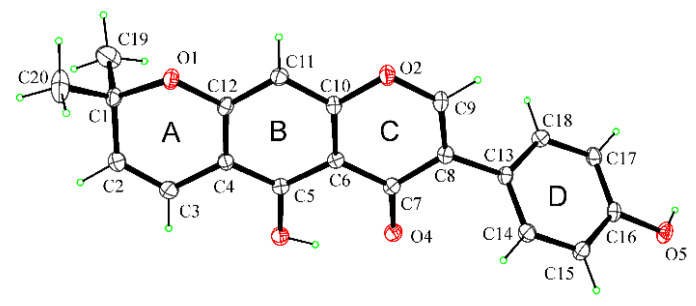
ORTEP view of alpinumisoflavone (**1**) with thermal ellipsoids drawn at the 30% probability level.

**Figure 4:**
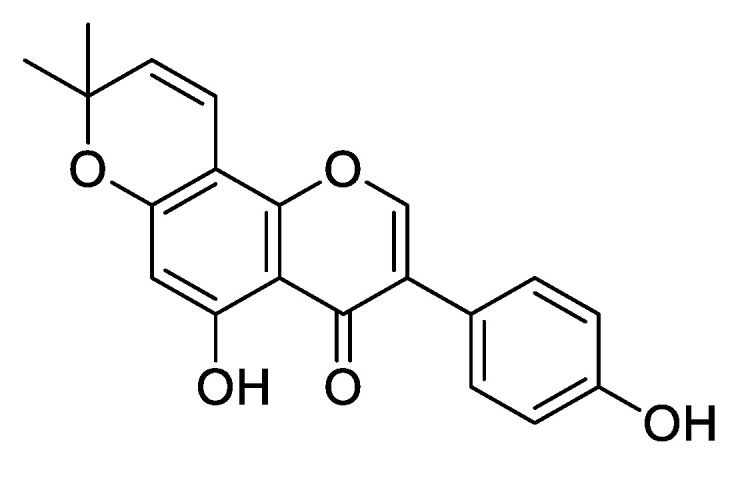
Structure of derrone, previously isolated from *R. raetam*.

The structure of alpinumisoflavone (**1**) was confirmed by X-ray diffractometric analysis. An ORTEP view is reported in Figure 3. The X-ray crystal structure is reported here to undoubtedly identify the compound **1** as alpinumisoflavone [45]. Alpinumisoflavone consists of three nearly coplanar fused rings, and one attached out-of-plane twisted phenyl ring. In the tricyclic ring system, a liner junction of A/B/C rings is observed. It should be noted that compound **1** showed a different junction between the dimethylpyran ring (A) and the chromenone moiety (B/C), with respect to the analog derrone (Figure 4) previously isolated from *R. raetam* flowers [46].

Although compounds **2**, **3**, and **6** have chiral centers, their absolute configurations were not determined so far. Due to the limited available amounts of these compounds, a modified Mosher method [47] was applied only to compound **6** to determine the absolute configuration of its secondary hydroxylated carbon (C-2”). When compound **6** was treated with S-MTPA chloride, its ester derivative showed two sets of signals with an enantiomeric ratio of 50:50, indication that **6** is an enantiomeric mixture of 2”(*S*)-**6** and 2”(*R*)-**6**. The same result was obtained when **6** was treated with *R*-MTPA chloride. An optical rotation value of zero [α]_D_^25^ 0 (c 0.4, MeOH) was also obtained. This result was not unexpected because a similar prenylated xantone, named (±)-graciesculenxanthone C, isolated from *Garcinia esculenta* showed an enantiomeric ratio of 60:40 when it was treated with *S*- and *R*-MTPA chloride [48].

**Figure 5:**
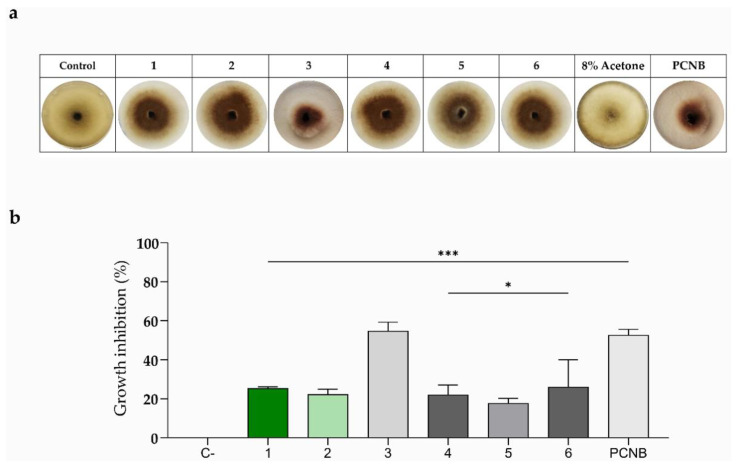
Effects of alpinumisoflavone (**1**), hydroxyalpinumisoflavone (**2**), laburnetin (**3**), licoflavone C (**4**), retamasin B (**5**), and ephedroidin (**6**) against *S. vesicarium*. The plot shows the fungal growth inhibition exerted by the tested compounds at a concentration of 50 µg/mL. (**a**) Representative photos of the biological assay for in vitro inhibition of mycelial growth of *S. vesicarium*. 8% Acetone and 0.5 mg/mL PCNB were used as negative and positive controls, respectively. (**b**) Fungal growth inhibition reported as the percentage of the reduction in the diameter of the fungal mycelium in the treated plate compared to that in the control plate. *** *p* < 0.0001 and * *p* < 0.05.

Thus, the six compounds (**1**–**6**) isolated from *R. raetam* were spot-inoculated on PDA plates to test their antifungal activity against the fungal phytopathogen *S. vesicarium*. As shown in Figure 5, only laburnetin (**3**) exhibited quite a strong activity when spot-inoculated (50 µg/mL), inhibiting the growth of *S. vesicarium* by around 55%, confirming the antagonistic effect displayed by the most active fractions, which inhibited *S. vesicarium* mycelium’s growth by around 60% (Figure 1). Interestingly, the commercial fungicide PCNB, used at a concentration of 0.5 mg/mL, exhibited an antagonistic effect comparable to the one exerted by laburnetin used at a 10-fold lower concentration (50 µg/mL) (Figure 5b), demonstrating how natural compounds could represent an effective alternative to chemicals in the agricultural field. The other compounds instead displayed a fungal inhibition of around 25% (**1**, **6**), 20% (**2**, **4**), and 17% (**5**) (Figure 5b). Considering the results obtained, it is possible to ascribe to laburnetin the main role in the antagonistic effect exhibited by *R. raetam* extract against the fungus *S. vesicarium*.

The isoflavonoid laburnetin (**3**) has already been isolated from the *Genista* genus [41], as well as from other plants, and its antimicrobial activity was demonstrated [49]. To the best of our knowledge, here, the antifungal activity of this compound against *S. vesicarium* is being reported for the first time, showing that laburnetin could be proposed as a natural antagonist for the control of this phytopathogen that infests several important cultivated species.

*R. raetam* is an allelopathic plant species collected in the Saharan ecosystem from the Souf region in southeastern Algeria. Allelochemicals involved in plant–plant interactions are a potential source for alternative agrochemicals to solve the negative effects caused by synthetic herbicides. Thus, the six metabolites (**1**–**6**) were tested on two independent parasitic weed bioassays to discover potential activities of *R. raetam* metabolites on either suicidal stimulation or radicle growth inhibition of broomrapes. First, the germination induction effect of alpinumisoflavone, ephedroidin, hydroxyalpinoisolflavone, laburnetin, licoflavone C, and retamasin B was tested on seeds of four parasitic weed species, *Orobanche crenata*, *O. cumana*, *O. minor,* and *Phelipanche ramosa,* using in vitro germination bioassays. The synthetic germination stimulant GR24 used as a positive control induced germination levels of 53.2% ± 1.7%, 64.6% ± 1.8%, 91.7% ± 1.7%, and 94.2% ± 0.4% in *O. crenata*, *O. cumana*, *O. minor,* and *P. ramosa,* respectively. Null germination was observed when seeds of the broomrape species were treated with the negative control (distilled water) or with compounds **1**–**6**. The results obtained in the germination bioassay indicate that none of the metabolites isolated from the stem of *R. raetam* act as suicidal germination inducers of the broomrape species studied. Field application of inductors of suicidal germination of broomrape seeds in the absence of a specific host is a control strategy for obligate root parasitic weeds. In fact, the subsequent parasitic growth after germination leads to the death of the parasite due to nutrient starvation in the absence of host-derived nutrients [50].

**Figure 6:**
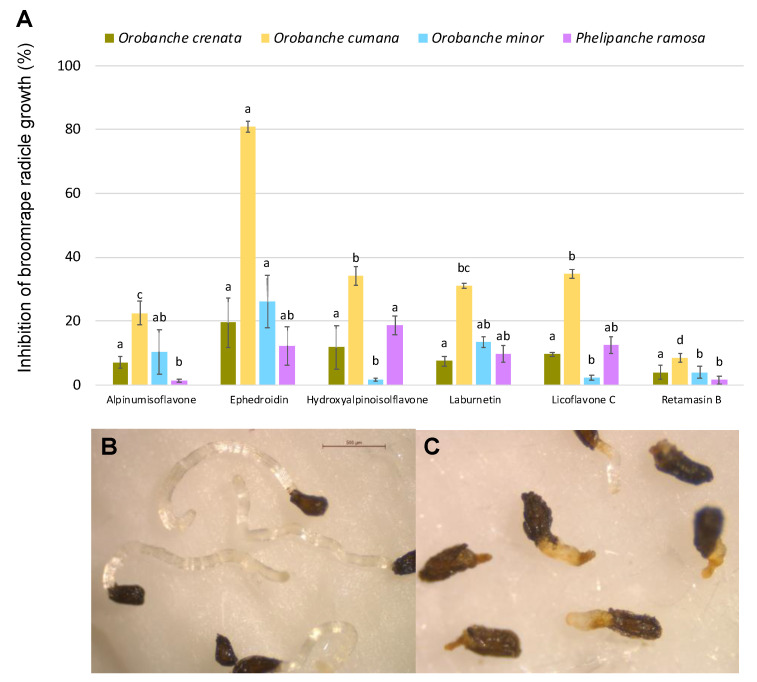
(**A**) Inhibition of broomrape radicle growth induced by alpinumisoflavone, ephedroidin, hydroxyalpinoisolflavone, laburnetin, licoflavone C, and retamasin B, expressed as a percentage with respect to the control GR24. (**B**,**C**) Photographs illustrating the effects of ephedroidin in radicles of *Orobanche cumana*: (**B**) control, (**C**) 100 μM ephedroidin. Analysis of variance was applied to angular transformed replicate data. For each broomrape species, bars with different letters are significantly different according to the Tukey test (*p* = 0.05). Error bars represent standard error.

In a second parasitic weed bioassay, the six purified compounds (**1**–**6**) were tested at 100 μM as potential inhibitors of radicle growth of *O. crenata, O. cumana*, *O. minor,* and *P. ramosa*. Among the compounds tested, low to negligible activity was found in all compounds tested except for ephedroidin (**6**), which strongly inhibited the normal development of *O. cumana* radicles in comparison with *O. cumana* control radicles (Figure 6A). Ephedroidin induced a strong toxic effect, observed as darkening in the *O. cumana* radicle and an average length inhibition of 80.8% ± 1.6% in comparison with radicles control (Figure 6B,C). Similar toxic effects on broomrape radicles were previously described for cytochalasans [51].

Ephedroidin (**6**) is a flavanoid previously isolated together with laburnetin (**3**) from the *Genista ephedroides* [41] and from *R. raetam* [52]. Recently, ephedroidin resulted to be the most active in inhibiting nitric oxide synthase (iNOS) and nuclear factor kappa B (NF-κB), as well as in decreasing oxidative stress, when compared with other flavonoids isolated from the same source [44]. Our results demonstrated that ephedroidin strongly inhibited the radical development of *O. cumana* seeds and can be proposed as a natural herbicide against this dangerous parasitic weed.

## 3. Conclusions

Bio-guided purification of *R. raetam* CH_2_Cl_2_ extract allowed us to isolate six metabolites, identified by spectroscopic and chemical methods as alpinumisoflavone, hydroxyalpinumisoflavone, laburnetin, licoflavone C, retamasin B, and efedroidin. In particular, the isoflavonoid laburnetin showed antifungal activity against the phytopathogen *S. vesicarium* 10-fold higher than that of the commercial fungicide PCNB. The flavonoid ephedroidin exhibited a strong inhibition of broomrape seed germination, suggesting their application as potential biopesticides against these noxious biotic stresses. Finally, the structure of alpinumisoflavone was confirmed by X-ray diffractometric analysis, which showed a different junction with respect to the analog derrone, previously isolated from the *R. raetam* plant. These data prompted further studies aimed to formulate the active compounds and test them in greenhouse and field trials. However, analyses on their ecotoxicological profile are needed before the practical application as biopesticides.

## 4. Materials and Methods

### 4.1. General Experimental Procedures

A JASCO P-1010 digital polarimeter was used to measure the optical rotations. A Bruker (Karlsruhe, Germany) spectrometer working at 400/100 MHz was used to record ^1^H/^13^C NMR spectra in CDCl_3_ or CD_3_OD, which were also used as internal standards. The LC/MS TOF system (Agilent 6230B, HPLC 1260 Infinity) (Milan, Italy) was used to record ESI mass spectra. Analytical and preparative Thin-Layer Chromatography (TLC) was performed on silica gel plates (Kieselgel 60, F_254_, 0.25 and 0.5 mm, respectively) (Merck, Darmstadt, Germany). The spots were visualized by exposure to UV light (254 nm) and/or iodine vapors and/or by spraying first with 10% H_2_SO_4_ in MeOH, and then with 5% phosphomolybdic acid in EtOH, followed by heating at 110 °C for 10 min. Column chromatography (CC) was performed using silica gel (Kieselgel 60, 0.063–0.200 mm) (Merck, Darmstadt, Germany). All the solvents were supplied by Sigma-Aldrich (Milan, Italy). The balance model used is Analytical ES 225SM-DR (Precisa, Dietikon, Switzerland).

### 4.2. Plant Material

Aerial parts of *R. raetam* were collected between December 2017 and February 2018, corresponding to the flowering phase of the plant. This study was carried out in the Souf region and is located in the north-east of Algerian Sahara, between 33° and 34° north latitude, and 6° and 8° longitude. Sea level = 40 m [32]. The plant material was then carefully rinsed with distilled water to remove dust particles and dried in the air for a few days at room temperature; finally, it was ground in a blender.

Seeds of parasitic weeds were collected from mature plants of *O. crenata* infecting pea in Spain, *O. cumana* infecting sunflower in Spain, *O. minor* infecting red clover in France, and *Phelipanche ramosa* infecting oilseed rape in France. Dry parasitic seeds were separated from capsules using winnowing combined with a sieve of 0.6 mm-mesh size and then stored dry in the dark at room temperature until use for this work.

### 4.3. Fungal Strain

The phytopathogen *S. vesicarium* was isolated from pears showing brown spot disease symptoms, sampled in Benevento, Campania, Italy, in 2019, as previously reported [1]. The strain was stored on Potato Dextrose Agar (PDA) plates in the culture collection of Agriges s.r.l., San Salvatore Telesino, Benevento, Italy (40.93345, 14.65799, 401 m.a.s.l.).

### 4.4. Extraction and Purification of Secondary Metabolites

Plant material (273.7 g) was extracted (1 × 500 mL) by H_2_O/MeOH (1/1, *v/v*), 1% NaCl, under stirred conditions at room temperature for 24 h, the suspension was centrifuged, and the supernatant was extracted by *n*-hexane (3 × 300 mL) and successively with CH_2_Cl_2_ (3 × 300 mL) and, after removing methanol under reduced pressure, with EtOAc (3 × 200 mL). The residue (6.7 g) of CH_2_Cl_2_ organic extract was purified by CC eluted with CH_2_Cl_2_/*i*-PrOH (9/1, *v/v*), yielding thirteen homogeneous fractions (F1–F13). The most active fractions (F2–F5, Figure 1) were further purified. In particular, the residue (70.2 mg) of F2 was purified by CC eluted with CH_2_Cl_2_/MeOH (97/3, *v/v*), yielding seven groups of homogeneous fractions (F2.1–F2.7). The residue (21.2 mg) of fraction F2.3 was further purified by TLC eluted with CH_2_Cl_2_/MeOH (97/3, *v/v*), yielding alpinumisoflavone (**1**, 12.9 mg). The residues of F3–F4 were combined (for a total amount of 235.6 mg) and then purified by CC eluted with CHCl_3_/*i*-PrOH (95/5, *v/v*), yielding seven fractions. The residue of the fourth fraction of the latter column were further purified by two successive steps on TLC eluted with CHCl_3_/MeOH (95/5, *v/v*) and EtOAc/*n*-hexane (4/6, *v/v*), yielding hydroxyalpinumisoflavone (**2**, 3.2 mg), laburnetin (**3**, 1.3 mg), and licoflavone C (**4**, 3.1 mg). The residue (98.2 mg) of F5 was purified by CC eluted with CH_2_Cl_2_/MeOH (95/5, *v/v*), yielding four fractions. The residue of the second fraction of this latter column was further purified by TLC eluted three times with acetone/*n*-hexane (4/6, *v/v*), yielding four metabolites. They were identified as retamasin B (**5**, 3.3 mg), ephedroidin (**6**, 10.1 mg), and further amounts of laburnetin (**3**, 1.0 mg, total of 2.3 mg) and licoflavone C (**4**, 1.1 mg, total of 4.2 mg).

Alpinumisoflavone (**1**): ^1^H and ^13^C NMR data are in agreement with those previously reported [38,39]. ESI MS (+) *m/z*: 695 [2M + Na]^+^, 337 [M + H]^+^.
Hydroxyalpinumisoflavone (**2**): ^1^H NMR data are in agreement with those previously reported [41]. ESI MS (+) *m/z*: 727 [2M + Na]^+^, 333 [M + H]^+^.
Laburnetin (**3**): ^1^H and ^13^C NMR data are in agreement with those previously reported [42]. ESI-MS (+), *m/z*: 355 [M + H]^+^.
Licoflavone C (**4**): ^1^H and ^13^C NMR data are in agreement with those previously reported [43]. ESI-MS (+), *m/z*: 339 [M + H]^+^.
Retamasin B (**5**): ^1^H and ^13^C NMR data are in agreement with those previously reported [44]. ESI-MS (+), *m/z*: 727 [2M + Na]^+^, 353 [M + H]^+^.
Ephedroidin (**6**): [α]_D_^25^ 0 (c 0.4, MeOH), ^1^H and ^13^C NMR data are in agreement with those previously reported [41]. ESI-MS (+), *m/z*: 355 [M + H]^+^.

### 4.5. X-ray Crystal Structure Analysis of Compound **1**

Single crystals of compound **1** suitable for X-ray analysis were obtained by slow evaporation from a mixture of MeOH:H_2_O (9.0:1.0). X-ray diffraction data were collected on a Bruker-Nonius KappaCCD diffractometer (Bruker-Nonius, Delft, The Netherlands) (graphite mono-chromated MoKα radiation, λ = 0.71073 Å). The structure was solved by direct methods (SIR97 program) [53] and anisotropically refined by the full-matrix least-squares method on F^2^ against all independent measured reflections (SHELXL-2018/3 program) [54]. H atoms of hydroxy groups were located in different Fourier maps and freely refined. All the other hydrogen atoms were introduced in calculated positions and refined according to the riding model. Platon TwinRotMap check suggests 2-axis (0 0 1) [1 0 4] twinning with basf 0.10, and refinement was performed using the HKLF5 data file. The figure of the ORTEP view was generated using the ORTEP-3 program [55].

Crystallographic Data of **1**: C_20_H_16_O_5_; *M*_r_= 336.33; monoclinic, space group *P*2_1_/c; *a* = 13.774(4) Å, *b* = 5.940(3)Å, *c =* 19.936(5) Å, *β* = 99.673(15)°; *V* = 1607.9(10) Å^3^; *T* = 173 K; *Z* = 4; *D*_c_ = 1.389 g cm^−3^; *μ* = 0.100 mm^−1^, *F* (000) = 704. Independent reflections: 9456. The final *R*_1_ values were 0.0569, w*R*_2_ = 0.1129 (*I* > 2*σ*(*I*)). Goodness of fit on F^2^ = 1.081. Largest diff. peak and hole = 0.203 and −0.252 e/Å^3^.

### 4.6. Antifungal Assay

The extract obtained from *R. raetam* aerial parts was tested against the phytopathogen *S. vesicarium,* as described by Yusoff et al. [56], with some modifications. The crude extract and the following fractions were dissolved in MeOH and mixed with 5 mL of cooled PDA to obtain a final concentration of 2 mg/mL and 250 µg/mL, respectively. The mix was then poured into Petri dishes and left to dry. Fungal plugs (6 × 6 mm diameter) cut from the growing edge of *S. vesicarium* mycelium were placed in the center of the plates and grown for 6/7 days at 28 ± 2 °C. Plates containing the fungal plugs alone were used as a control. As a positive control, fungicidal pentachloronitrobenzene ≥ 94% (PCNB) (Sigma-Aldrich, Saint-Louis, MO, USA) dissolved in toluene was used. Toluene and MeOH were used as negative controls. The in vitro antifungal bioassays of the purified metabolites, **1** laburnetin, **2** licoflavone C, **3** alpinumisoflavone, **4** hydroxyalpinumisoflavone, **5** raetamsin B, and **6** ephedroidin, were performed according to the method previously described in [57], with some modifications. The metabolites and PCNB dissolved in 8% acetone and toluene, respectively, were placed at the four opposite sides of each Petri dish, 1 cm away from the fungal plug at the center of the plate, at a final concentration of 50 µg/mL. Acetone and toluene were used as negative controls. The plates were incubated for 6/7 days at 28 ± 2 °C. The percentage of inhibition of the fungal growth was calculated using the following formula:

% = [(*Rc* − *Ri*)/*Rc*] × 100 (1)

where *Rc* is the radial growth of the test fungi in the control plates (mm), and *Ri* is the radial growth of the fungi in the presence of different compounds tested (mm). The results show the antifungal activity of different compounds analyzed by ANOVA using Tukey’s test. The experiments were performed in triplicate.

### 4.7. Broomrape Assays

Allelopathic effects of alpinumisoflavone, ephedroidin, hydroxyalpinoisolflavone, laburnetin, licoflavone C, and retamasin B were tested on broomrape suicidal germination and radicle growth in two independent bioassays conducted according to previous protocols [51].

Seeds of four broomrape species, *Orobanche crenata*, *Orobanche cumana*, *Orobanche minor,* and *Phelipanche ramosa,* were surface-sterilized by immersion in 0.5% (*w/v*) NaOCl and 0.02% (*v/v*) Tween 20, for 5 min, rinsed thoroughly with sterile distilled water, and dried in a laminar airflow cabinet. First, broomrape seeds were submitted to a conditioning period using a warm stratification, as follows. Approximately 100 seeds of each broomrape species were placed separately on 9 mm-diameter glass fiber filter paper disks (GFFP) (Whatman International Ltd., Maidstone, UK), moistened with 50 μL of sterile distilled water, and placed in incubators at 23 °C for 10 days inside Parafilm-sealed Petri dishes, to allow seed conditioning.

Then, GFFP disks containing conditioned broomrape seeds were transferred onto a sterile sheet of filter paper and transferred to new 9 cm sterile Petri dishes. For the assay of suicidal germination, induction stock solutions of each metabolite respectively dissolved in methanol were individually diluted in sterile distilled water up to an equivalent concentration of 100 μM. For the assay of radicle growth inhibition, stock solutions of each metabolite respectively dissolved in methanol were individually diluted to 100 μM using an aqueous solution of GR24. For each assay, triplicate aliquots of each sample were applied to GFFP discs containing conditioned broomrape seeds. Treated seeds were incubated in the dark at 23 °C for 7 days and the percent of germination and radicle growth was determined for each GFFP disc, as described previously [51], using a stereoscopic microscope (Leica S9i, Leica Microsystems GmbH, Wetzlar, Germany). For germination induction assays, the germination was determined by counting the number of germinated seeds on 100 seeds for each GFFP disk. For the characteristic of radicle growth, the value used was the average of 10 randomly selected radicles per GFFP disc [58]. The percentage of germination induction of each metabolite was then calculated relative to the average germination of control seeds (seeds treated with water), and the percentage of radicle growth inhibition of each treatment was then calculated relative to the average radicle growth of control treatment (radicles treated with GR24) [41].

### 4.8. Data Analysis

Statistical analyses were performed using GraphPad Prism 8 software, and data were expressed as the mean ± SD. Differences among groups were compared by the ANOVA test. Differences were considered statistically significant at *p* < 0.05.

## Data Availability

Not applicable.

## Supplementary Materials

The following supporting information can be downloaded at: https://www.mdpi.com/article/10.3390/toxins14050311/s1, Scheme S1:Extraction and bioguided purification of compounds 1–6 from R. raetam aerial parts; Figure S1: ^1^H NMR spectrum of alpinumisoflavone, 1 (acetone-*d6*, 400 MHz); Figure S2: ^13^C NMR spectrum of alpinumisoflavone, 1 (acetone-*d6*, 100 MHz); Figure S3: ESI MS spectrum of alpinumisoflavone, 1 recorded in positive modality; Figure S4: ^1^H NMR spectrum of hydroxyalpinumisoflavone, 2 (MeOD, 500 MHz); Figure S5: ESI MS spectrum of hydroxyalpinumisoflavone, 2 recorded in positive modality; Figure S6: H NMR spectrum of laburnetin, 3 (MeOD, 500 MHz); Figure S7: ESI MS spectrum of laburnetin, 3 recorded in positive modality; Figure S8: ^1^H NMR spectrum of licoflavone C, 4 (acetone-*d6*, 500 MHz); Figure S9: ESI MS spectrum of licoflavone C, 4 recorded in positive modality; Figure S10: ^1^H NMR spectrum of retamasin B, 5 (acetone-*d6*, 400 MHz); Figure S11: ^13^C NMR spectrum of retamasin B, 5 (acetone-*d6*, 100 MHz); Figure S12: ^13^C NMR spectrum of retamasin B, 5 (acetone-*d6*, 100 MHz); Figure S13: ^1^H NMR spectrum of ephedroidin, 6 (acetone-*d6*, 500 MHz); Figure S14: ESI MS spectrum of ephedroidin, 6 recorded in positive modality.
