# Supplementary material for: Specialized Metabolites from the Allelopathic Plant Retama raetam as Potential Biopesticides
Source: Toxins (Basel). 2022 Apr 28;14(5):311. doi: 10.3390/toxins14050311 (PMC9146260; doi:10.3390/toxins14050311)
Supplement: Supplementary file 1 [file toxins-14-00311-s001.zip › toxins-1696863-supplementary.pdf]

toxins

Supplementary Materials: Specialized Metabolites from the Allelopathic Plant *Retama raetam* as Potential Biopesticides

Gabriele Soriano [1], Claudia Petrillo [2,*], Marco Masi [1,*], Mabrouka Bouafiane [3,4], Aminata Khelil [3], Angela Tuzi [1], Rachele Isticato [2], Mónica Fernández-Aparicio [5,†] and Alessio Cimmino [1,†]

[1] Department of Chemical Sciences, University of Naples Federico II, 80126 Naples, Italy; gabriele.soriano@unina.it (G.S.); angela.tuzi@unina.it (A.T.); alessio.cimmino@unina.it (A.C.)
[2] Department of Biology, University of Naples Federico II, 80126 Naples, Italy; isticato@unina.it
[3] Laboratoire de Protection des Ecosystèmes en Zones Arides et Semi-Arides, Universit'e Kasdi Merbah-Ouargla, Ouargla 30000, Algeria; bouafiane-mabrouka@univ-eloued.dz (M.B.); aminatakhelil@yahoo.fr (A.K.)
[4] Department of Agronomy, Faculty of Life and Natural Sciences, University of El Oued, El Oued 39000, Algeria
[5] Department of Plant Breeding, Institute for Sustainable Agriculture (IAS), CSIC, Avenida Menéndez Pidal s/n, 14004 Córdoba, Spain; monica.fernandez@ias.csic.es
* Correspondence: claudia.petrillo@unina.it (C.P.); marco.masi@unina.it (M.M.).
† These authors contributed equally to this work.

Abstract: To cope with the rising food demand, modern agriculture practices are based on the indiscriminate use of agrochemicals. Although this strategy leads to a temporary solution, it also severely damages the environment, representing a risk to human health. A sustainable alternative to agrochemicals is the use of plant metabolites and plant-based pesticides, known to have minimal environmental impact compared to synthetic pesticides. *Retama raetam* is a shrub growing in Algeria's desert areas, where it is commonly used in traditional medicine because of its antiseptic and antipyretic properties. Furthermore, its allelopathic features can be exploited to effectively control phytopathogens in the agricultural field. In this study, six compounds belonging to isoflavones and flavones subgroups have been isolated from the *R. raetam* dichloromethane extract and identified using spectroscopic and optical methods as alpinumisoflavone, hydroxyalpinumisoflavone, laburnetin, licoflavone C, retamasin B, and ephedroidin. Their antifungal activity was evaluated against the fungal phytopathogen *Stemphylium vesicarium* using a growth inhibition bioassay on PDA plates. Interestingly, the flavonoid laburnetin, the most active metabolite, displayed an inhibitory activity comparable to that exerted by the synthetic fungicide pentachloronitrobenzene, in a ten-fold lower concentration. The allelopathic activity of *R. raetam* metabolites against parasitic weeds was also investigated using two independent parasitic weed bioassays to discover potential activities on either suicidal stimulation or radicle growth inhibition of broomrapes. In this latter bioassay, ephedroidin strongly inhibited the growth of *Orobanche cumana* radicles and, therefore, can be proposed as a natural herbicide.

Keywords: biocontrol; *Retama raetam*; *Stemphylium vesicarium*; *Orobanche cumana*; laburnetin; ephedroidin

Supporting Information List

Page 3: **Scheme S1.** Extraction and bioguided purification of compounds **1-6** from *R. raetam* aerial parts.

Page 3: **Figure S1**. ^{1}H NMR spectrum of alpinumisoflavone, **1** (acetone-d_6, 400 MHz).

Page 4: **Figure S2**. ^{13}C NMR spectrum of alpinumisoflavone, **1** (acetone-d_6, 100 MHz).

Page 4: **Figure S3**. ESI MS spectrum of alpinumisoflavone, **1** recorded in positive modality.

Page 5: **Figure S4**. ^{1}H NMR spectrum of hydroxyalpinumisoflavone, **2** (MeOD, 500 MHz).

Page 5: **Figure S5.** ESI MS spectrum of hydroxyalpinumisoflavone, **2** recorded in positive modality.

Page 6: **Figure S6**. ^{1}H NMR spectrum of laburnetin, **3** (MeOD, 500 MHz).

Page 6: **Figure S7**. ESI MS spectrum of laburnetin, **3** recorded in positive modality.

Page 7: **Figure S8**. ^{1}H NMR spectrum of licoflavone C, **4** (acetone-d_6, 500 MHz).

Page 7: **Figure S9**. ESI MS spectrum of licoflavone C, **4** recorded in positive modality.

Page 8: **Figure S10**. ^{1}H NMR spectrum of retamasin B, **5** (acetone-d_6, 400 MHz).

Page 8: **Figure S11**. ^{13}C NMR spectrum of retamasin B, **5** (acetone-d_6, 100 MHz).

Page 9: **Figure S12.** ESI MS spectrum of retamasin B, **5** recorded in positive modality.

Page 9: **Figure S13**. ^{1}H NMR spectrum of ephedroidin, **6** (acetone-d_6, 500 MHz).

Page 10: **Figure S14**. ESI MS spectrum of ephedroidin, **6** recorded in positive modality.

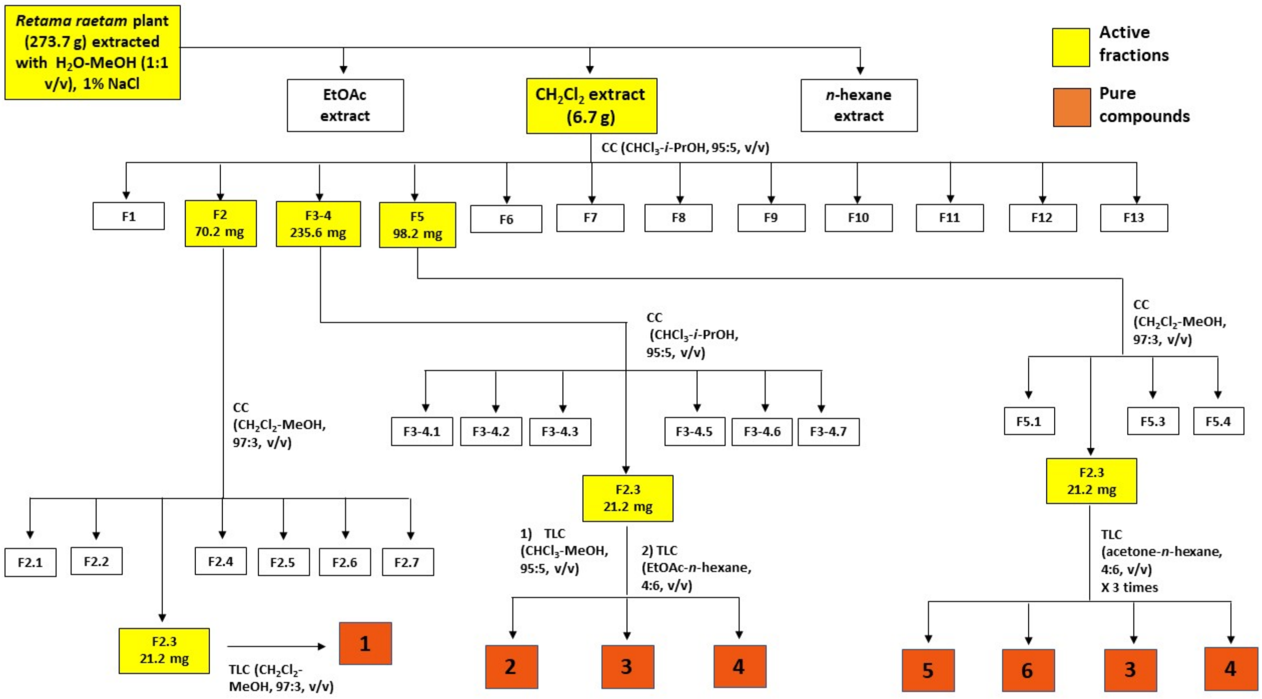

Scheme S1. Extraction and bioguided purification of compounds **1-6** from *R. raetam* aerial parts.

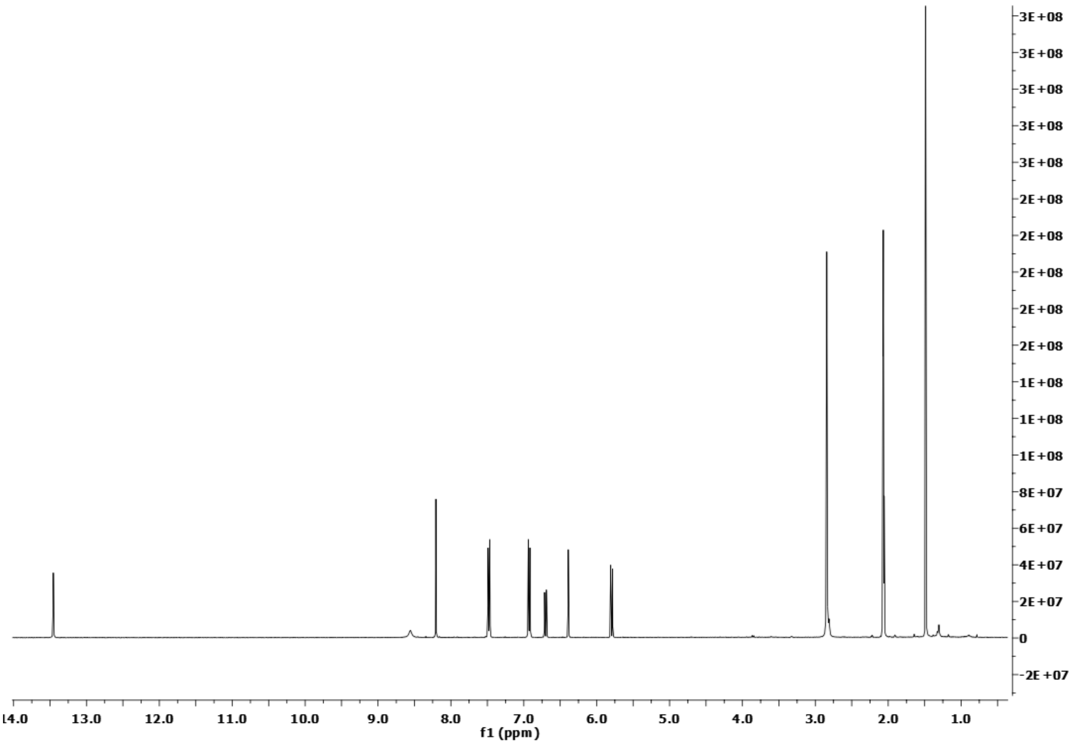

Figure S1. ^1H NMR spectrum of alpinumisoflavone, **1** (acetone-d_6, 400 MHz).

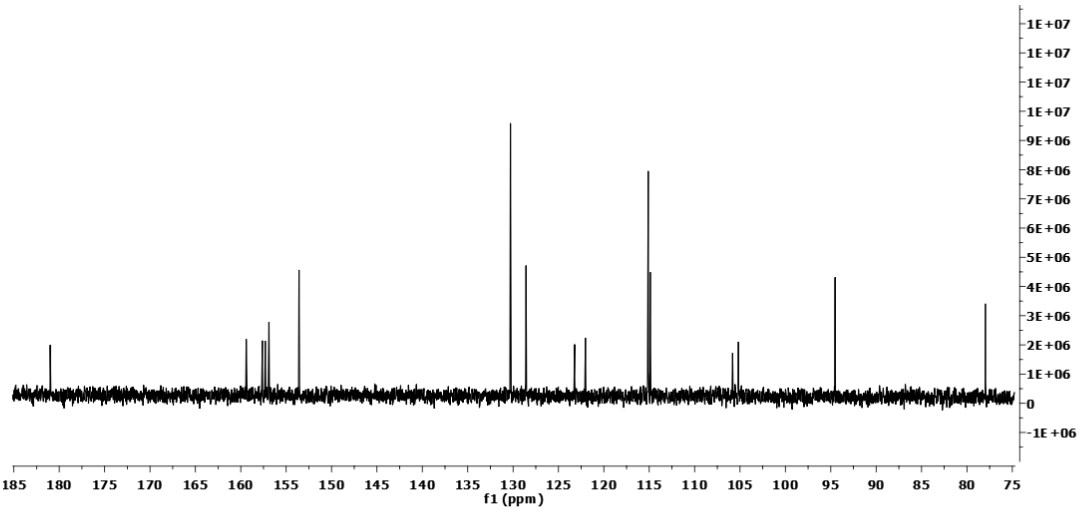

Figure S2. ^{13}C NMR spectrum of alpinumisoflavone, **1** (acetone-d_6, 100 MHz).

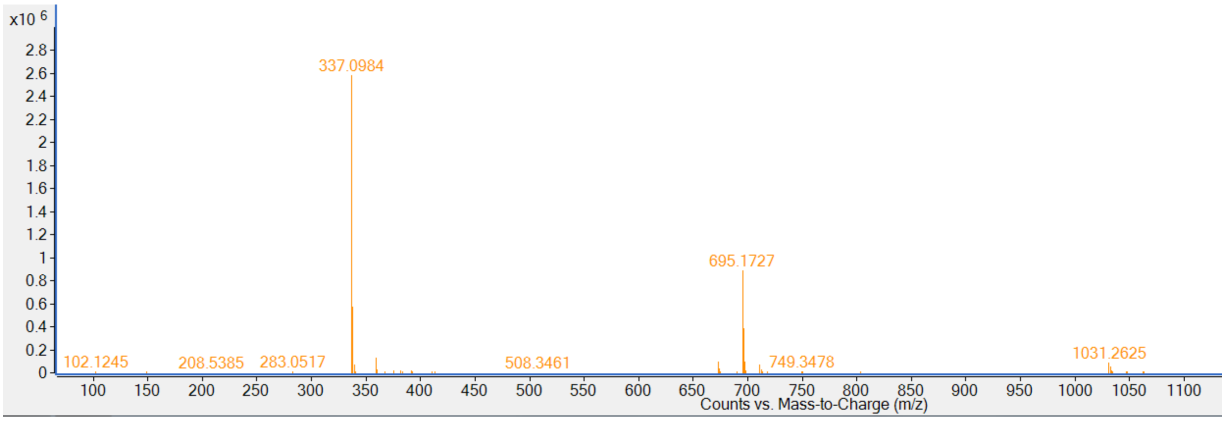

Figure S3. ESI MS spectrum of alpinumisoflavone, **1** recorded in positive modality.

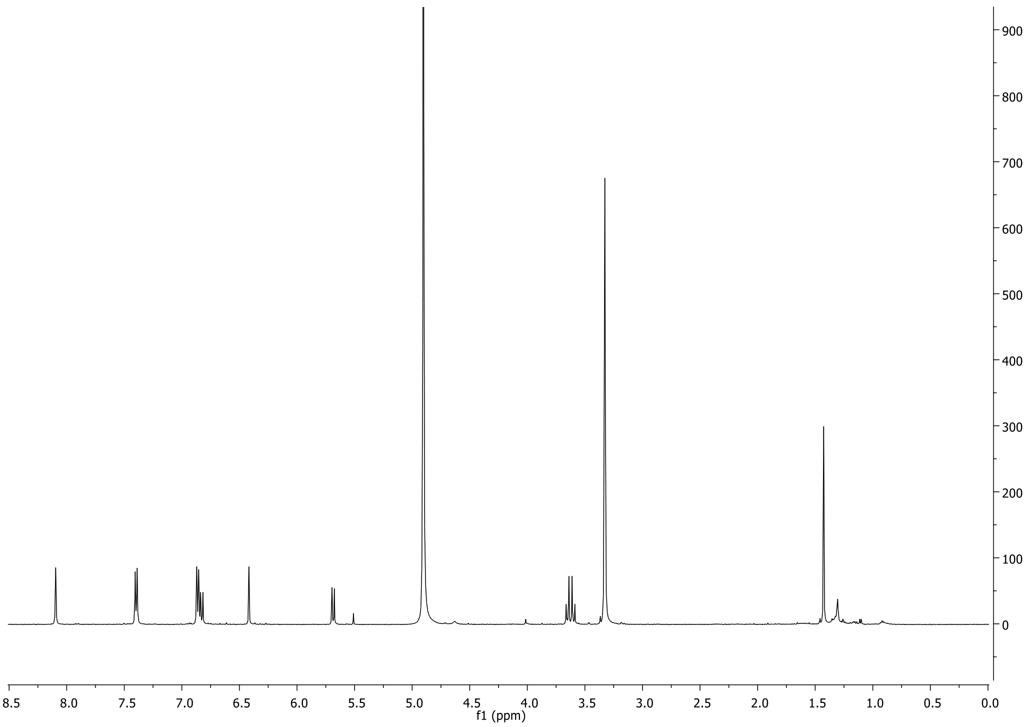

Figure S4. ^{1}H NMR spectrum of hydroxyalpinumisoflavone, **2** (acetone-d_6, 400 MHz).

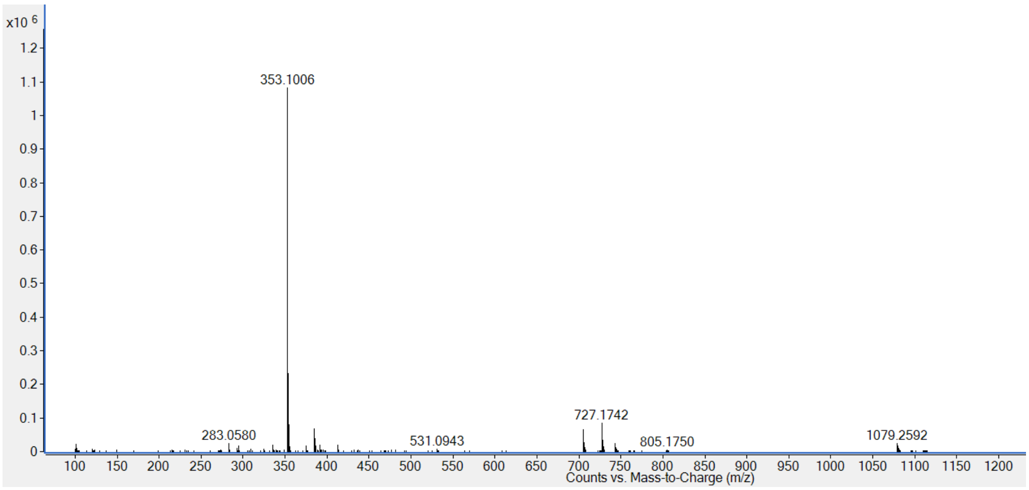

Figure S5. ESI MS spectrum of hydroxyalpinumisoflavone, **2** recorded in positive modality.

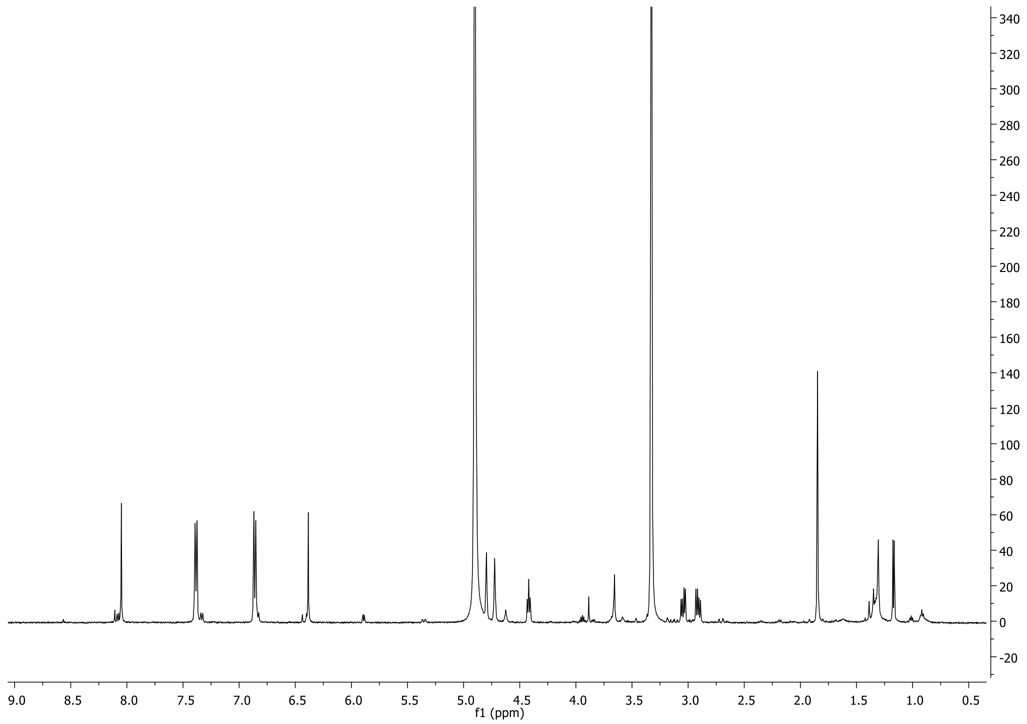

Figure S6. ^{1}H NMR spectrum of laburnetin, **3** (acetone-d_6, 400 MHz).

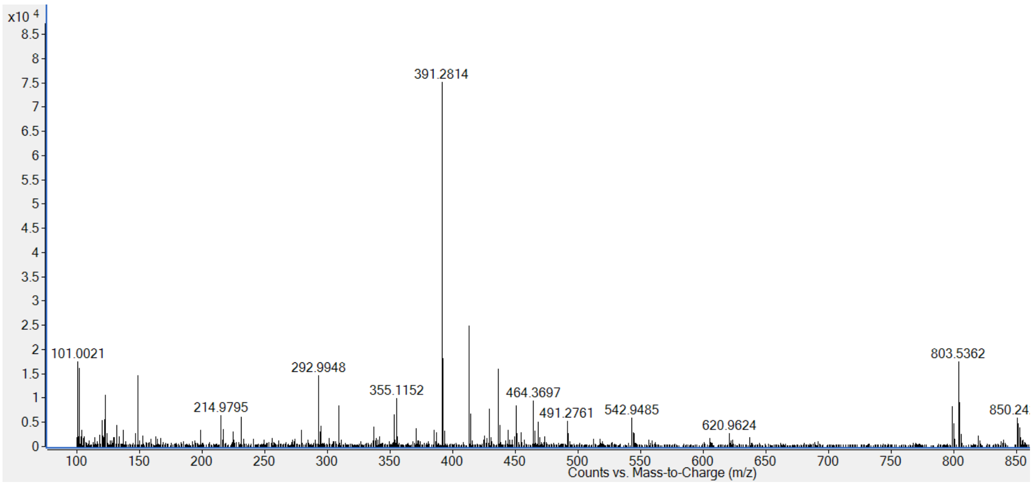

Figure S7. ESI MS spectrum of laburnetin, **3** recorded in positive modality.

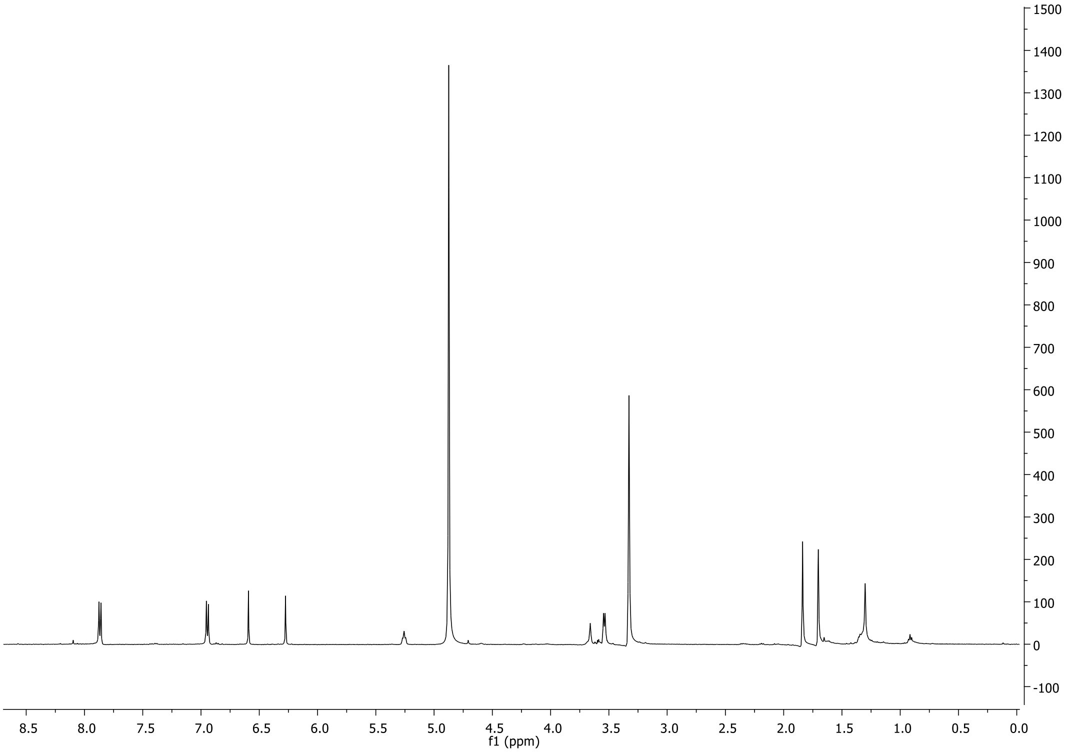

Figure S8. ^{1}H NMR spectrum of licoflavone C, **4** (acetone-d_6, 400 MHz).

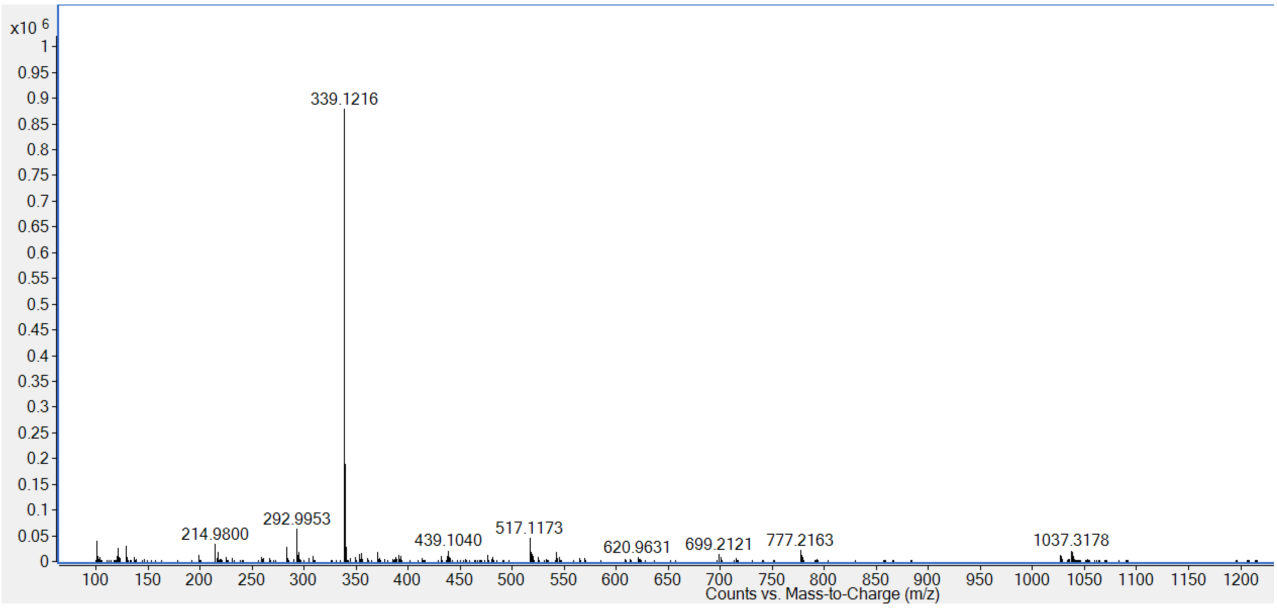

Figure S9. ESI MS spectrum of licoflavone C, **4** recorded in positive modality.

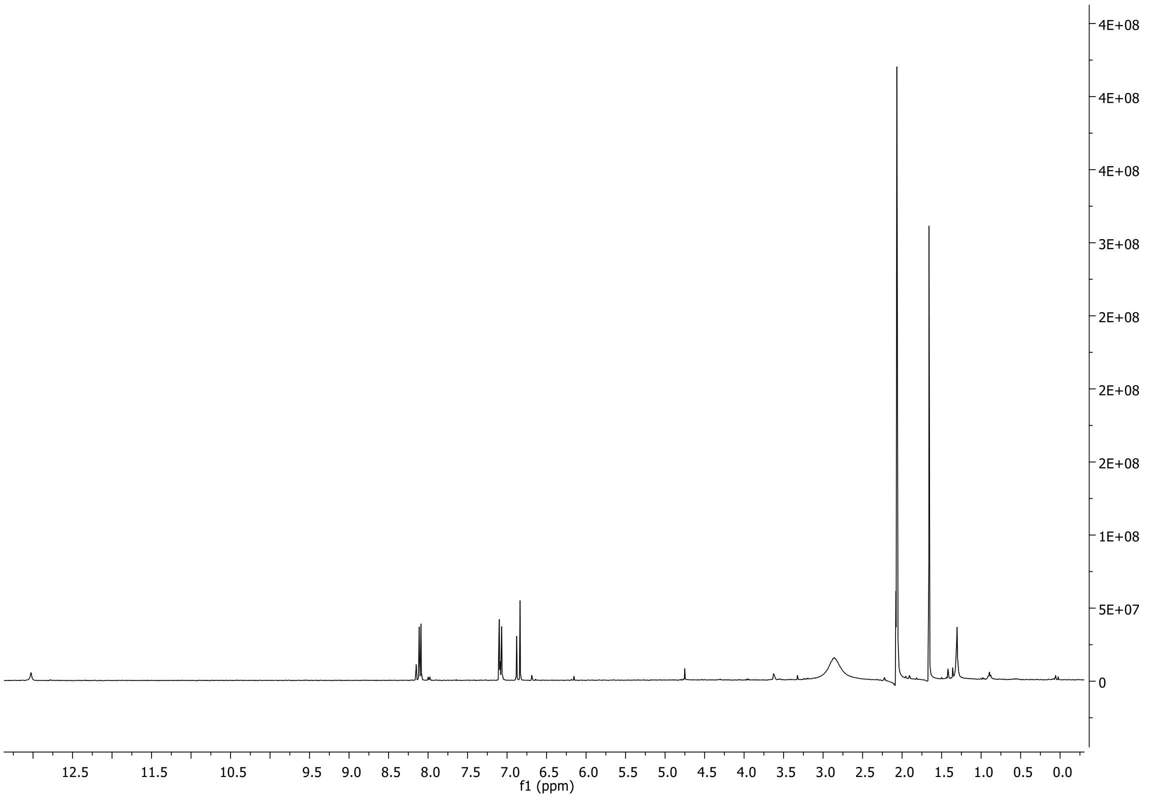

Figure S10. ^{1}H NMR spectrum of retamasin B, **5** (acetone-d_6, 400 MHz).

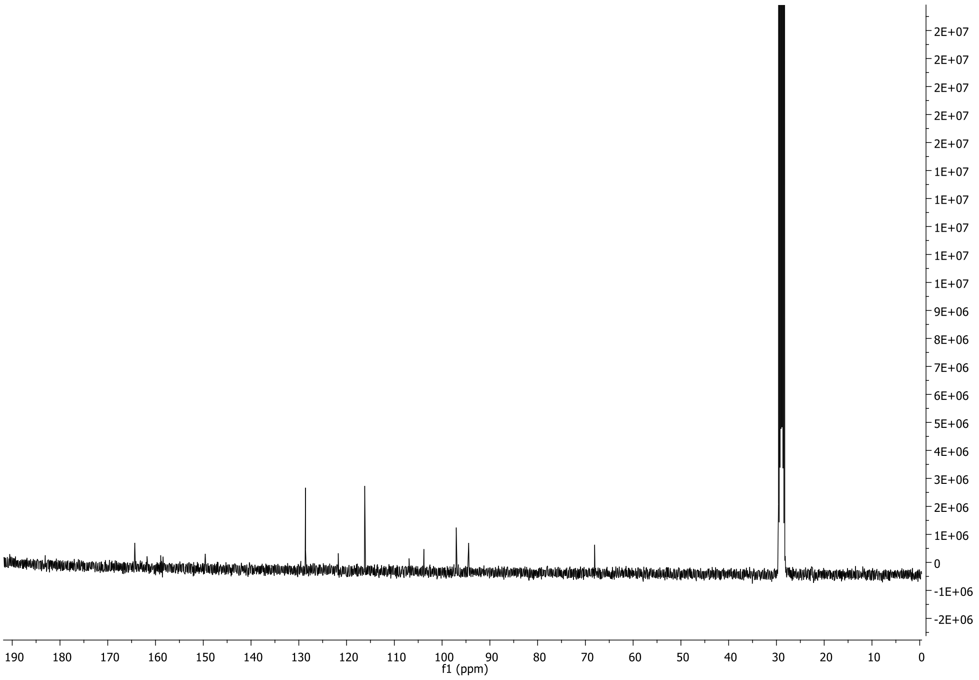

Figure S11. ^{13}C NMR spectrum of of retamasin B, **5** (acetone-d_6, 100 MHz).

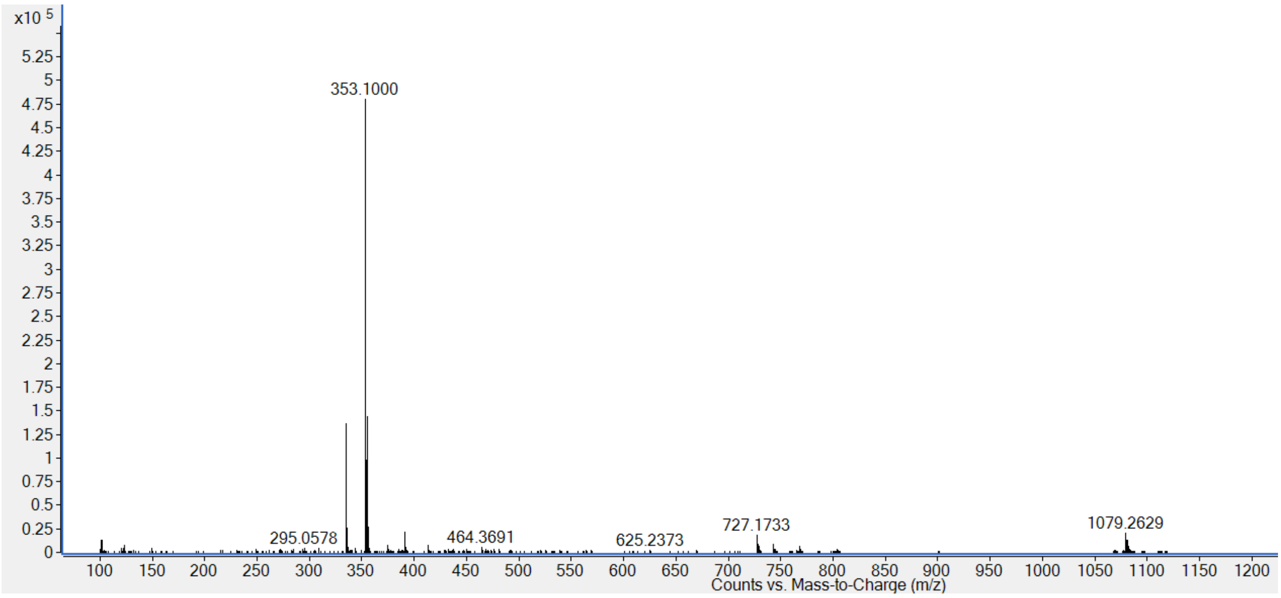

Figure S12. ESI MS spectrum of retamasin B, **5** recorded in positive modality.

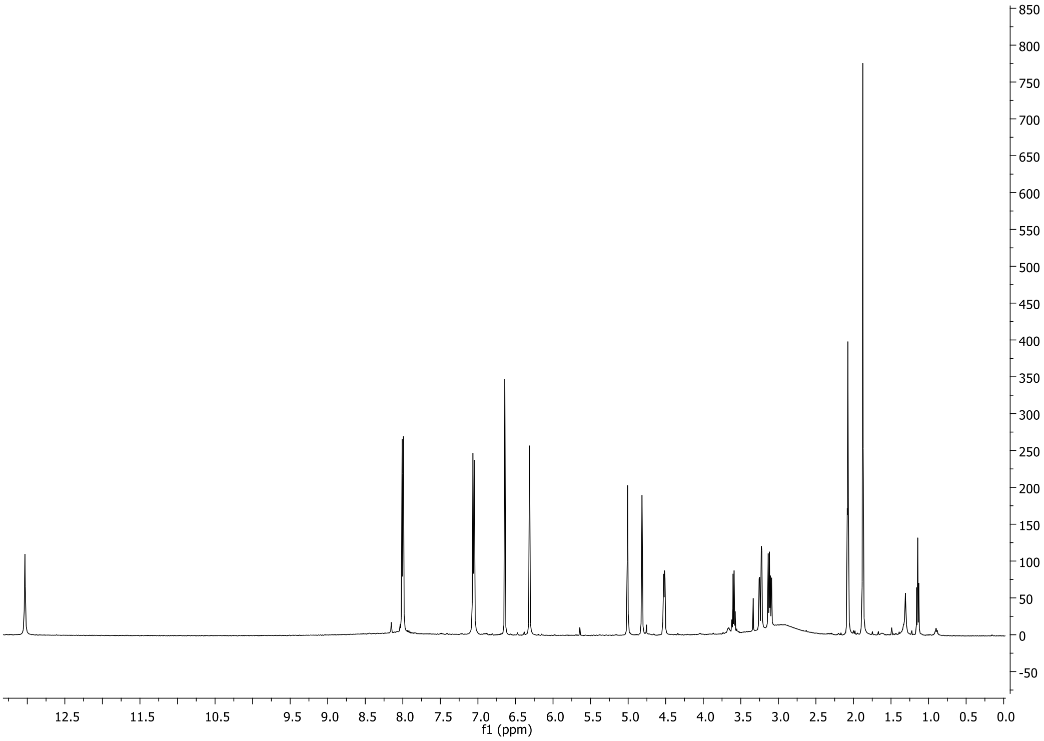

Figure S13. ^{1}H NMR spectrum of ephedroidin, **6** (acetone-d_6, 400 MHz).

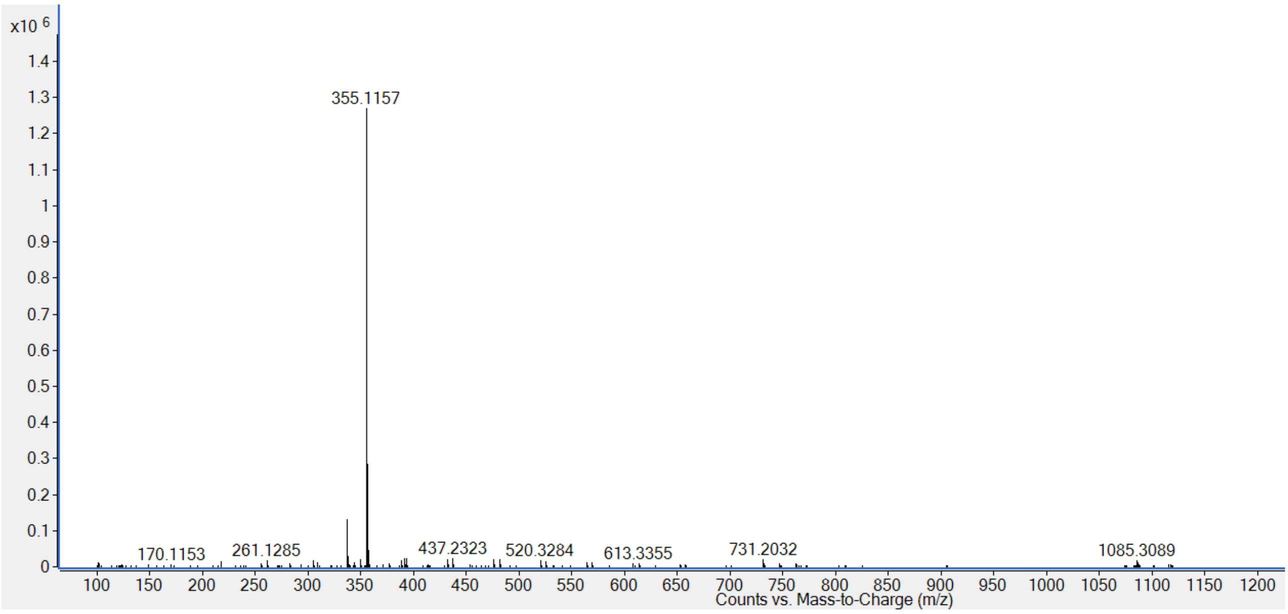

Figure S14. ESI MS spectrum of ephedroidin, **6** recorded in positive modality.
